# Assessing therapeutic alliance and client satisfaction across teletherapy, in-person, and hybrid modalities in clients with depression and anxiety disorders: a cross-sectional study

**DOI:** 10.25122/jml-2025-0113

**Published:** 2025-11

**Authors:** Amal Ibrahim Khalil, Amnah Jambi, Haneen Fahad Alsulami, Jawnahthamer Althagafi, Renad Taher Emmam

**Affiliations:** 1 King Abdullah International Medical Research Center, Jeddah, Saudi Arabia; 2 King Saud bin Abdulaziz University for Health Sciences, College of Nursing, Jeddah, KSA; 3 Ministry of National Guard Health Affairs (MNGHA); 4 Menoufyia University, Faculty of Nursing, Shebin El Kom, Egypt

**Keywords:** teletherapy, face-to-face therapy, therapeutic alliance, client satisfaction, hybrid, preference, employment, gender, Saudi context, KAIMRC, King Abdullah International Medical Research Centre, IRB, Institutional Review Board, MOH, Ministry of Health, WAI-SR, Working Alliance Inventory - short revised, CSQ-8 Client Satisfaction Questionnaire (8 items), ANOVA, Analysis of Variance, LSD, Least Significant Difference, COVID-19, Coronavirus Disease 2019, CBT, Cognitive Behavioral Therapy, DSM-5, Diagnostic and Statistical Manual of Mental Disorders; Fifth Edition, PTSD, Post-Traumatic Stress Disorder

## Abstract

The swift growth of teletherapy has sparked discussions about its effectiveness in comparison to traditional in-person therapy, especially in building a robust therapeutic alliance among those with anxiety and depressive disorders. The study aimed to compare the therapeutic alliance and client satisfaction across teletherapy, in-person, and hybrid modalities in the management of depression and anxiety disorders. A cross-sectional study was conducted using snowball sampling, involving 377 participants from various cities in Saudi Arabia, with a significant proportion residing in the Makkah Region (79.6%). The sample was predominantly female (69.5%), single (60.2%), and young adults aged 18–24 (53.1%). Three validated instruments were used: the Working Alliance Inventory-Short Revised (WAI-SR) to measure therapeutic alliance, and the Client Satisfaction Questionnaire (CSQ-8) to assess satisfaction. ANOVA results indicated no statistically significant difference in WAI-SR scores among the treatment groups (face-to-face, remote, and hybrid) (F = 2.804, *P* = .062), suggesting a similar therapeutic alliance across the modalities. However, ANOVA revealed significant differences in client satisfaction by therapy type, though further post hoc analyses are needed to identify specific group differences. Preferences varied: 52.5% favored face-to-face therapy, 24.1% preferred teletherapy, and 23.3% had no choice. Both face-to-face and teletherapy are effective in establishing a strong therapeutic alliance. Given the notable differences in satisfaction levels, mental health services should consider individual preferences to enhance treatment engagement and outcomes.

## Introduction

In recent years, technological advancements have transformed global mental health care delivery, with teletherapy emerging as a prominent mode of remote intervention [1]. This highlights the need to evaluate how teletherapy compares with traditional face-to-face (FTF) therapy in treating depression and anxiety disorders as classified in the Diagnostic and Statistical Manual of Mental Disorders, Fifth Edition (DSM-5), focusing on therapeutic alliance and patient satisfaction within Saudi Arabia’s cultural context [2,3].

Major depressive disorder (MDD) and anxiety disorders are among the leading causes of disability worldwide, with their burden increasing significantly since the onset of the COVID-19 pandemic. Global analyses estimate that the pandemic alone led to approximately 26 million new cases of MDD and 28 million new cases of anxiety in 2020 [4]. Reflecting these global trends, a national survey reported a rise in mental health concerns and a growing demand for psychological services, particularly among young people and working adults [5]. In response to this growing need, teletherapy has become a viable alternative to traditional mental health care for individuals. International meta-analyses have confirmed that remote psychological interventions for depression and anxiety are as effective as direct care to reduce symptoms [6]. Similarly, studies conducted in Saudi Arabia during and after the outbreak found that telehealth played an important role in ensuring access to mental health services, especially in geographically underserved areas such as the northern border and southern provinces [7]. Despite cultural norms that can affect communication and disclosure, Saudi clients generally show openness to virtual therapy when the needs of technology and privacy are met [8]. The core of effective therapy, regardless of the method, is the therapeutic alliance, which refers to the emotional connection, mutual trust, and shared goals of cooperation between the therapist and the client. The evidence shows that the strength of this alliance is among the strongest predictors of positive outcomes in psychotherapy. Although teletherapy supports the development of a strong alliance, a 2024 meta-analysis found slightly weaker overlap and outcome correlations than in personal therapy, suggesting potential challenges to relationships in digital environments [9]. However, comparative studies, including those in the Gulf region, show that online alliances can be compared in quality to those constructed.

Client satisfaction also plays an essential role in facilitating engagement and continuity in therapy. In Saudi Arabia, customers are generally satisfied with telemental health services, but when people have experience with both formats, satisfaction with telemental health is slightly lower than with in-person therapy [10]. Factors contributing to this include digital literacy, the therapist's ability to respond, and perceptions of privacy and confidentiality.

A recent local study found that customers who used hybrid services were most satisfied, as they could build initial relationships in person and later maintain them online [11]. Hybrid or hybrid care models, which integrate in-person sessions with structured online components, are increasingly popular globally and nationwide. Mixed cognitive-behavioral therapy (b-CBT) is not inferior to traditional CBT in both clinical efficacy and clinical involvement [12,13].

Preliminary results from the Saudi pilot project conducted at the university counselling center suggest that hybrid models can enhance alliances and satisfaction by balancing flexibility with face-to-face connection [14-16]. Despite these developments, limited research has systematically compared therapeutic alliances with client satisfaction across teletherapy, in-person, and hybrid modalities. Understanding these dynamics is crucial to designing effective and culturally sensitive mental health services after the epidemic in Saudi Arabia. Therefore, this cross-sectional study examined and compared the quality of therapeutic alliances and levels of client satisfaction among individuals diagnosed with depression or anxiety receiving treatment through one of the three modalities. The findings aim to provide insights to guide the development of future mental health service models within the evolving global landscape.

### Significance of the study

The rapid growth of teletherapy has transformed mental health service delivery by enhancing accessibility and flexibility, particularly for individuals facing logistical, geographic, or stigma-related barriers to care [17]. In Saudi Arabia, depression and anxiety are significant public health concerns, with recent studies showing prevalence rates ranging from 20% to over 40% in the general population, and even higher among specific groups such as students and healthcare workers. These mental health disorders greatly contribute to psychological distress, reduced quality of life, and increased healthcare utilization. To meet the rising demand for mental health services, Saudi Arabia has implemented telemental health services, offering over 38,000 online psychological consultations from 2020 to 2021. Despite this expansion, questions remain about whether teletherapy can foster the same level of therapeutic alliance and client satisfaction as in-person psychotherapy [6,18].

The therapeutic alliance, a crucial component of effective therapy and treatment adherence, may be influenced by the unique communication styles and relational nuances present in virtual versus face-to-face settings [7]. Similarly, client satisfaction is an essential indicator of perceived care quality and a predictor of long-term engagement and treatment success [19].

Given the high prevalence of depression and anxiety disorders and the increasing adoption of teletherapy and hybrid models in Saudi Arabia's healthcare system, it is vital to examine how different therapy formats affect the therapeutic relationship and satisfaction outcomes. This understanding will support evidence-based planning and guide the optimization of mental health service delivery in hybrid or post-pandemic environments [20].

### Theoretical framework

**Figure 1:**
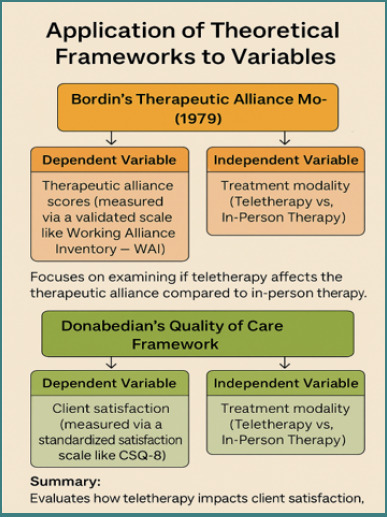
Theoretical framework application to the dependent and independent variables of the study

This research is grounded in Bordin’s Therapeutic Alliance Model [21], which defines the therapeutic relationship as consisting of three essential elements: consensus on treatment objectives, allocation of therapeutic tasks, and the establishment of a personal connection between the client and therapist. These components are crucial for achieving positive results in the treatment of anxiety and depression. They could be affected by how therapy is delivered, whether through teletherapy or in-person sessions [7]. Moreover, Donabedian’s Quality of Care Framework [22] was utilized to evaluate client satisfaction, representing the outcome of care influenced by both the structure (e.g., a telehealth platform or a physical clinic) and the process (the interaction between the therapist and the client). This dual-framework method facilitates an in-depth understanding of how the treatment modality influences both the quality of the therapeutic relationship and client-perceived satisfaction, two key measures of mental healthcare effectiveness [6,19]. Figure 1 illustrates and summarizes the application of two theories on the dependent, independent variables, the outcome (client satisfaction), and the process (therapy modalities).

## Material and Methods

### Study design

A cross-sectional comparative correlational survey was selected because it permits the simultaneous assessment of key variables (therapeutic alliance and client satisfaction) in a large number of respondents without the expense or delay associated with follow-up designs [23]. Cross-sectional studies are widely regarded as economical and time-efficient approaches for estimating prevalence and exploring associations in mental health research.

### Setting

Data were gathered nationwide to maximize geographical spread and ecological validity. Recruitment links were disseminated through licensed mental health professionals who provided both face-to-face and teletherapy across all five principal regions of Saudi Arabia, with the largest share of respondents (43%) residing in Makkah Province.

### Participants

Eligible participants were adults (≥ 18 years) with a clinician-confirmed diagnosis of a depressive or anxiety disorder who were currently engaged in either synchronous teletherapy (video or audio) or traditional in-person psychotherapy.

Exclusion criteria were acute psychosis, suicidal crisis, or any condition requiring immediate psychiatric intervention, as these circumstances may impair the capacity to give informed consent or distort alliance and satisfaction ratings.

### Sampling and sample size

Because mentalhealth help-seeking can be stigmatized, a non-probability snowball approach was used: initial clients and treating clinicians forwarded the survey link to peers, thereby extending reach into otherwise hidden networks. Snowball sampling is a practical method for accessing hard-to-reach populations and is particularly appropriate when studying sensitive mental health topics [24].

For a conservative population estimate of 20,000 adult therapy clients, Rao Soft’s online calculator (5 % margin of error, 95 % confidence level) indicated a minimum of 377 respondents (Rao Soft Inc., 2004), a target we exceeded.

### Study tools (data collection instruments)

Three main instruments were employed to collect data for the present study, as outlined below:

1. **A demographic characteristics tool** that collected data on age, gender, marital status, type of therapy, etc.
2. **The Client Satisfaction Questionnaire (CSQ-8)**, developed by Larsen *et al*. [25], is a widely used instrument for assessing client satisfaction in mental health services. It is typically self-administered and completed after therapy sessions. The CSQ-8 consists of 8 items without subscales, each rated on a 4-point Likert scale from 1 (Poor) to 4 (Excellent), yielding a total score of 8-32, with higher scores indicating greater satisfaction. Scoring ranges from 8 to 32, with higher scores indicating greater satisfaction. Although all items are positively worded, items 2, 4, 5, and 8 are reverse-scored to minimize response bias. The CSQ-8 has demonstrated high reliability and validity, as confirmed by its original developers and subsequent studies [26,27]. Its proven internal consistency, test–retest reliability, and concurrent validity make it a robust and versatile instrument for use in both clinical practice and research settings across diverse populations.
3. **The Working Alliance Inventory Short Form Revised (WAI-SR)** was used as a condensed version of a 36-item tool developed by Adam Harvath [28] in 1989, drawing on Brodin's [29] alliance model from 1979. Comprising 12 items, the WAI-SR offers a more time-efficient assessment, making it particularly suitable for repeated use in clinical and research settings. It evaluates three key aspects of therapeutic alliance: (a) agreement on treatment goals, (b) alignment on treatment tasks, and (c) the quality of the bond between patient and therapist. Responses are provided on a Likert scale ranging from 1 to 5, with higher scores indicating a stronger alliance. The subscale scores range from 5 to 20, with specific items allocated to each subscale. Notably, the WAI-SR demonstrates high internal consistency and reliability, as evidenced by robust Cronbach alpha scores ranging from 0.81 to 0.90 for subscales and 0.91 for the total score. Its construct validity is further supported by strong correlations with other established alliance measures, such as the Helping Alliance Questionnaire and the California Psychotherapy Alliance Scale (Cronbach's alpha = 0.90) [30].

### Arabic translation and cultural adaptation

Both instruments are accessible in Arabic. The WAI website features an approved Arabic short-form translation, while the CSQ-8 has been translated into over 55 languages, including Arabic [27,28]. The World Health Organization (WHO) guidelines for the translation and adaptation of measures were adhered to, which encompass:

- Initial translation by two bilingual psychologists.
- Reconciliation by an expert panel to ensure semantic and conceptual equivalence.
- Independent blind back-translation by a linguist.
- Pre-testing with 30 clients, followed by a cognitive debriefing to verify clarity.
- Pilot internal consistency was excellent (CSQ8 α = 0.88; WAISR α = 0.89).

### Data collection procedure

This cross-sectional study collected data from July 2024 to April 2025 in several cities throughout Saudi Arabia, with many participants originating from the Makkah Province, including Jeddah, Makkah, and Taif. A non-probability snowball sampling method was used, in which initial participants were invited to join the study and subsequently asked to recommend other eligible individuals from their personal or professional circles.

Ethical approval was obtained from the appropriate Institutional Review Board (IRB), and all participants provided informed consent, either electronically or in writing, before participating. To be included, participants had to be at least 18 years old, diagnosed with depression and/or an anxiety disorder, and undergoing either teletherapy or in-person psychotherapy. Eligibility required completion of at least three therapy sessions in their current treatment format. Those receiving both therapy types simultaneously or with severe psychiatric comorbidities (e.g., psychosis or bipolar I disorder) were excluded. Participants were categorized into the teletherapy or in-person therapy group based on their treatment method. Data were collected via online survey forms (e.g., Google Forms) for teletherapy participants and via paper- or tablet-based forms at clinic locations for in-person participants. Each participant completed the following assessments:

The Working Alliance Inventory – Short Form (WAI-SF) to evaluate the therapeutic alliance and the CSQ-8 to gauge therapy satisfaction. In addition, participants completed a demographic and clinical questionnaire that collected information on variables including age, gender, education level, diagnosis, therapy duration, and the number of sessions attended. The entire survey required approximately 15–20 minutes to complete. Trained research assistants were available, either virtually or onsite, to offer support and ensure data quality. All responses were anonymized and stored in a secure, password-protected database, accessible only to the principal investigators and research team.

### Data management and analysis

The data were coded and analyzed using the latest version of the Statistical Package for the Social Sciences (SPSS). Descriptive statistics—including frequencies, percentages, means, and standard deviations—were used to summarize the study variables. The Chi-squared (χ^2^) test or Fisher’s exact test was employed to compare categorical variables, while the Student’s *t*-test and analysis of variance (ANOVA) were used to examine differences between numerical and categorical variables. The Pearson correlation coefficient was used to assess the strength and direction of associations among the studied variables. A *P* value of less than 0.05 (*P* < 0.05) was considered statistically significant.

## Results

Table 1 presents the sociodemographic and clinical characteristics of the study participants. The majority were men (69.5%), single (60.2%), and residing in urban areas (79.6%), with a substantial proportion holding postgraduate degrees (64.7%). The prevalence of self-employment was notably high (49.1%), suggesting a possible connection between job autonomy and experiences related to mental health. Anxiety was more common (60.2%) than depression (39.8%), with generalized anxiety disorder being the most frequently identified specific diagnosis (38.7%). Treatment preferences showed a strong inclination towards therapy (52.5%), with combined therapy (38.2%) outpacing solitary medication treatment (29.4%), highlighting a nuanced perspective on mental health care. The age distribution leaned towards younger individuals, with 53.1% in the 18–24 age range, which may indicate an early onset of mental health difficulties or a greater tendency to seek help among younger individuals.

**Table 1:** Distribution of participants according to their demographic characteristics, *n* = 377

Characteristic	Category	Frequency (*n* = 377)	Percentage (%)
**Gender**	Male	262	69.5
	Female	115	30.5
**Marital status**	Single	227	60.2
	Married	140	37.1
	Divorced	6	1.6
	Widows	4	1.1
**Age group**	18–24 years	200	53.1
	25–34 years	53	14.1
	35–44 years	57	15.1
	45–54 years	51	13.5
	55–64 years	11	2.9
	65+ years	5	1.3
**Education level**	Primary	7	1.9
	Secondary	10	2.7
	Undergraduate	110	29.2
	Postgraduate	244	64.7
	Doctorate	6	1.6
**Employment status**	Unemployed	117	31.0
	Part-time	10	2.7
	Full-time	54	14.3
	Self-employed	185	49.1
	Retired	11	2.9
**Residency area**	Urban	300	79.6
	Suburban	30	8.0
	Rural	47	12.4
**General diagnosis**	Depression	150	39.8
	Anxiety	227	60.2
**Specific diagnosis**	Generalized Anxiety Disorder	146	38.7
	Panic Disorder	51	13.5
	Obsessive-Compulsive Disorder	30	8.0
	Major Depressive Disorder	104	27.6
	Postpartum Depression	17	4.5
	Premenstrual Dysphoric Disorder	29	7.7
**Therapy type**	Psychotherapy	122	32.4
	Medication	111	29.4
	Combined Therapy	144	38.2
Preference	Therapy	198	52.5
	Medication	91	24.1
	No preference	88	23.3

Table 2 presents the results of the CSQ scale, indicating a generally positive evaluation of the mental health services provided. A majority of participants rated the service as 'Good' or 'Excellent' across most items. Specifically, 58.4% of respondents rated the overall quality of service as 'Excellent', while 53.6% reported that their needs were met at a 'Good' level, and an additional 30.5% selected 'Excellent'. Satisfaction levels were also high, with 39.5% of participants rating it 'Excellent', though 22.0% rated it 'Fair'. Recommendation rates were strong, with nearly half (48.3%) rating the service as 'Excellent' and 40.3% rating it as 'Good'. In terms of helpfulness, 38.2% believed the service addressed their concerns, while 39.0% were fully confident in its effectiveness. General satisfaction was widely positive, with 47.5% feeling 'Very satisfied' and 44.8% reporting they were 'Mostly satisfied'. Improvement in participants' situations was also notable, with 44.6% stating the service helped them 'a great deal'. The likelihood of future use was promising, with 42.7% of respondents indicating they would use the service again. However, a segment of participants, ranging between 16.4% and 22.0% across items, reported dissatisfaction or limited effectiveness, suggesting potential areas for improvement, particularly in meeting individualized concerns and expectations.

**Table 2:** CSQ full scale with corresponding statements and participants' response distributions, *n* = 377

CSQ Item	Statement	Response Options	Frequency (*n* = 377)	Percentage (%)
**CSQ1**	How would you rate the quality of service you received?	Poor	3	0.8
		Fair	45	11.9
		Good	109	28.9
		Excellent	220	58.4
**CSQ2**	Did the service meet your needs?	Poor	13	3.4
		Fair	47	12.5
		Good	202	53.6
		Excellent	115	30.5
**CSQ3**	How satisfied were you with the service?	Poor	9	2.4
		Fair	83	22.0
		Good	136	36.1
		Excellent	149	39.5
**CSQ4**	Would you recommend this service to others?	Poor	9	2.4
		Fair	34	9.0
		Good	152	40.3
		Excellent	182	48.3
**CSQ5**	Was the service helpful in addressing your concerns?	No, not	14	3.7
		No, I do not think so	72	19.1
		Yes, I think so	144	38.2
		Yes, definitely	147	39.0
**CSQ6**	Overall, how satisfied are you with your experience?	Quite dissatisfied	2	0.5
		Indifferent or mildly dissatisfied	27	7.2
		Mostly satisfied	169	44.8
		Very satisfied	179	47.5
**CSQ7**	Did the service improve your situation?	No, they seemed to make things worse	6	1.6
		No, they did not help	62	16.4
		Yes, they helped somewhat	141	37.4
		Yes, they helped a great deal	168	44.6
**CSQ8**	Would you use this service again?	No, not	19	5.0
		No, I do not think so	43	11.4
		Yes, I think so	154	40.8
		Yes, definitely	161	42.7

Table 3 presents the WAI scale results, indicating a strong therapeutic alliance between participants and their therapists, with consistently high ratings across key relationship indicators. Agreement on therapy goals was well-established, as 79.9% reported aligning with their therapist 'Fairly often' to 'Always'. Collaborative engagement in therapy sessions was also prominent, with 57.1% experiencing teamwork 'Very often' or 'Always'. Comfort in discussing concerns was substantial, with 61.8% feeling at ease 'Very often' or 'Always', while 65% believed their therapist understands their problems well. Notably, emotional connection was strong, as over half (52.3%) reported always feeling a strong connection to their therapist. Support and guidance from therapists received high scores, with trust levels at 69.8% for 'Very often' or 'Always'. Progress in therapy was reflected in 65% of participants perceiving meaningful improvement, and attentive listening was also well-rated, with 64.8% feeling heard. However, small segments of respondents indicated lower agreement or collaborative experiences, suggesting room for improvement in tailored therapeutic approaches for some individuals

**Table 3:** WAI scale distribution among participants of the study, *n* = 377

WAI Item	Statement	Seldom	Sometimes	Fairly Often	Very Often	Always
**WAI1**	The therapist and I agree on the goals of therapy	16 (4.2%)	57 (15.1%)	109 (28.9%)	116 (30.8%)	79 (21.0%)
**WAI2**	We work collaboratively in therapy sessions	14 (3.7%)	69 (18.3%)	79 (21.0%)	122 (32.4%)	93 (24.7%)
**WAI3**	I feel comfortable discussing concerns with my therapist	12 (3.2%)	47 (12.5%)	85 (22.5%)	126 (33.4%)	107 (28.4%)
**WAI4**	My therapist understands my problems	8 (2.1%)	51 (13.5%)	73 (19.4%)	109 (28.9%)	136 (36.1%)
**WAI5**	I feel connected to my therapist	8 (2.1%)	23 (6.1%)	55 (14.6%)	94 (24.9%)	197 (52.3%)
**WAI6**	My therapist supports me in therapy	6 (1.6%)	40 (10.6%)	77 (20.4%)	93 (24.7%)	161 (42.7%)
**WAI7**	I believe my therapist values my feelings	10 (2.7%)	36 (9.5%)	74 (19.6%)	108 (28.6%)	149 (39.5%)
**WAI8**	The therapist and I share a common understanding of my goals	8 (2.1%)	36 (9.5%)	67 (17.8%)	119 (31.6%)	147 (39.0%)
**WAI9**	The therapist and I collaborate effectively	11 (2.9%)	44 (11.7%)	89 (23.6%)	115 (30.5%)	118 (31.3%)
**WAI10**	I trust my therapist’s guidance	5 (1.3%)	38 (10.1%)	71 (18.8%)	121 (32.1%)	142 (37.7%)
**WAI11**	My therapist helps me make meaningful progress	10 (2.7%)	34 (9.0%)	88 (23.3%)	122 (32.4%)	123 (32.6%)
**WAI12**	My therapist listens to my concerns attentively	10 (2.7%)	31 (8.2%)	92 (24.4%)	122 (32.4%)	122 (32.4%)

**Figure 2:**
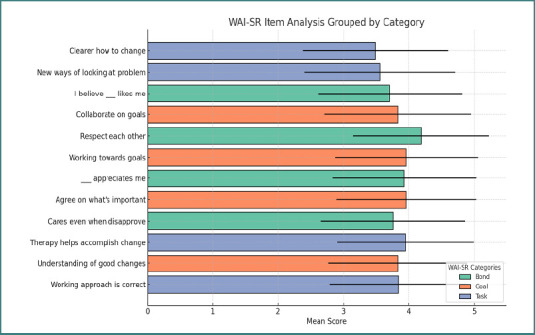
Working alliance inventory categories score among participants

Figure 2 shows the Working Alliance Inventory Short Revised (WAI-SR) results, indicating a strong therapeutic bond, with the highest mean score (4.19) reflecting mutual respect between participants and therapists. The goal dimension also demonstrated positive engagement, with goal-setting items scoring an average of around 3.9, indicating shared collaboration. Task-related items had slightly lower mean scores (3.5–3.9), suggesting that while tasks were perceived as beneficial, there may be room for improved clarity. Table 4 presents the chi-square test results (χ^2^ = 39.26, *P* < 0.00001), indicating a statistically significant association between treatment type and participant preference. This suggests that preferences for therapy modalities do not align proportionally with the actual treatment choices observed. Notably, face-to-face therapy had a higher expected preference (52.5%) than its observed usage (32.4%), suggesting that more participants would have preferred this modality than those who used it. Conversely, remote therapy had a slightly lower preference expectation (24.1%) than its observed usage (29.4%), suggesting some participants may have used it despite a lower overall preference. The category of 'Both/No preference' showed a substantial discrepancy, with a higher observed treatment rate (38.2%) compared to its preference expectation (23.3%), indicating that many participants in this group may have had practical considerations influencing their choice rather than a strong preference.

**Table 4:** Distribution of participants according to their treatment types and their preferences, *n* = 377

Category	Observed (Treatment)		Expected (Preference)		Chi-square test	*P* value
**Face-to-face therapy**	122	32.4	198	52.5	χ^2^ = 39.26	< 0.00001
**Remote therapy**	111	29.4	91	24.1		
**Hybrid/No preference**	144	38.2	88	23.3		

The Pearson correlation coefficient between the Total Working Alliance Inventory (WAI-12) score and the Client Satisfaction Questionnaire (CSQ-8) score was 0.649, which was statistically significant at the 0.01 level (*P* = .000; Table 5). This indicates a moderate to strong positive relationship between the two variables. In other words, as the quality of the working alliance between clients and providers increased, client satisfaction also tended to increase. This finding underscores the significance of the therapeutic alliance in promoting client satisfaction within clinical or counseling settings.

**Table 5:** Pearson correlation between working alliance (WAI-12) and client satisfaction (CSQ-8) among study participants, *n* = 377

Variables	Total WAI-12	Total CSQ-8
**Total WAI-12**	1.000	.649**
**Total CSQ-8**	.649**	1.000
**Sig. (2-tailed)**	—	.000
***n***	377	377

Note: Correlation was significant at the 0.01 level (2-tailed)

As shown in Table 6, several demographic variables had significant effects on client satisfaction (CSQ-8) but had weaker effects on the therapeutic alliance (WAI-12), with medium to large effect sizes (η^2^ = 0.06–0.09) for gender, marital status, age, and employment status. Female participants, married individuals, and employed individuals reported greater satisfaction with their therapy experience, consistent with previous research indicating that women and married clients may feel more emotionally supported and experience greater relational consistency during therapy. In contrast, employed clients may feel more satisfied due to perceived increased autonomy and stability. Education level did not differ significantly.

**Table 6:** One-way ANOVA and effect size results comparing CSQ-8 and WAI-12 scores, *n* = 377

Comparison	Sum of squares (between groups)	df	Mean square	F Value	*P* value (Sig.)	Effect size (η^2^)
**CSQ-8 × Gender**	255.080	1	255.080	10.996	0.001	0.029
**WAI-12 × Gender**	490.959	1	490.959	4.468	0.035	0.012
**CSQ-8 × Marital Status**	754.664	3	251.555	11.444	0.000	0.084
**WAI-12 × Marital Status**	719.554	3	239.851	2.183	0.090	0.017
**CSQ-8 × Age Group**	787.707	5	157.541	7.157	0.000	0.088
**WAI-12 × Age Group**	934.317	5	186.863	1.701	0.133	0.013
**CSQ-8 × Education Level**	64.753	4	16.188	0.677	0.608	0.007
**WAI-12 × Education Level**	600.801	4	150.200	1.360	0.247	0.012
**CSQ-8 × Employment**	563.266	4	140.816	6.243	0.000	0.062
**WAI-12 × Employment**	1336.170	4	334.042	3.079	0.016	0.033

Effect size η^2^ (partial eta squared) interpretation: small = .01, moderate = .06, large = .14. CSQ-8: Client Satisfaction Questionnaire; WAI-12: Working Alliance Inventory.

On the other hand, most demographic factors had little to no impact on the therapeutic alliance, and there were no discernible differences in education level (WAI-12). This supports the idea that interpersonal and therapeutic factors, rather than demographics, are what largely determine the therapist-client relationship. It shows that alliance quality stayed largely constant across gender, age, and socioeconomic groups.

Client satisfaction (CSQ-8) varied significantly across therapy modalities, as indicated in Table 7. Compared with those who received only remote therapy (*P* < 0.001) or face-to-face therapy (*P* < 0.001), participants who received both face-to-face and remote (hybrid) therapy reported significantly higher satisfaction. The moderate effect sizes (η^2^ = 0.082–0.086) suggest that the type of therapy had a significant impact on satisfaction levels. On the other hand, there was no discernible difference between in-person and remote therapy alone (*P* = 0.956), suggesting that these two single-modality approaches were equally satisfying. These results imply that the hybrid therapy model provides the best possible balance between the flexibility of teletherapy and the relational depth of face-to-face interaction. This combination seems to improve overall satisfaction, perhaps by allowing for continuity of care while considering clients' comfort and scheduling preferences.

**Table 7:** ANOVA multiple comparisons among types of therapies and participants’ preferences, *n* = 377

(I) Treatment type	(J) Treatment type	Mean Difference (I–J)	Std. Error	Sig.	Effect size (η^2^)
Face-to-face therapy	Remote therapy	-0.034	0.627	0.956	0.021
Face-to-face therapy	Both	-2.190*	0.588	0.000	0.086
Remote therapy	Face-to-face therapy	0.034	0.627	0.956	0.021
Remote therapy	Both	-2.156*	0.603	0.000	0.082
Both	Face-to-face therapy	2.190*	0.588	0.000	0.086
Both	Remote therapy	2.156*	0.603	0.000	0.082

* . The mean difference is significant at the 0.05 level. Effect size η^2^ (partial eta squared) interpretation: small = .01, moderate = .06, large = .14. Dependent Variable: CSQ-8 Satisfaction Post Hoc Test: LSD

Table 8 shows no statistically significant differences in satisfaction (CSQ-8) or therapeutic alliance (WAI-12) across general or specific diagnostic categories (*P* > 0.05), with very small effect sizes (η^2^ ≤ 0.02) indicating minimal influence of diagnosis on therapy perceptions. Therapy type had a significant effect on satisfaction (CSQ-8: F = 9.218, *P* < 0.001, η^2^ = 0.047), suggesting that modality (face-to-face, teletherapy, hybrid) accounts for approximately 4.7% of the variance in satisfaction, representing a small-to-moderate practical impact. In contrast, therapeutic alliance did not differ significantly by therapy type (*P* = 0.062), implying that rapport and engagement were maintained across modalities. Therapy preference did not significantly affect satisfaction or alliance (*P* > 0.05), although small effect sizes (η^2^ = 0.009–0.012) suggest subtle trends that may be relevant in larger samples.

**Table 8:** ANOVA results – influence of diagnosis, therapy type, and therapy preference on CSQ-8 and WAI-12 scores, *n* = 377

Comparison	Sum of squares (between groups)	df	Mean square	F Value	*P* value (Sig.)	Effect size (η^2^)
**CSQ-8 × General Diagnosis**	9.576	1	9.576	0.401	0.527	0.001
**WAI-12 × General Diagnosis**	0.053	1	0.053	0.000	0.983	0.000
**CSQ-8 × Specific Diagnosis**	168.606	5	33.721	1.424	0.215	0.019
**WAI-12 × Specific Diagnosis**	655.680	5	131.136	1.186	0.316	0.016
**CSQ-8 × Therapy Type**	420.659	2	210.330	9.218	0.000	0.047
**WAI-12 × Therapy Type**	615.877	2	307.939	2.804	0.062	0.015
**CSQ-8 × Therapy Preference**	109.572	2	54.786	2.317	0.100	0.012
**WAI-12 × Therapy Preference**	373.976	2	186.988	1.693	0.185	0.009

Table 9 indicates that therapy type had a significant effect on client satisfaction (CSQ-8) scores (F = 9.218, *P* < .001), while its impact on the working alliance (WAI-12) was borderline and not statistically significant (*P* = .062). Neither general nor specific diagnoses significantly influenced CSQ-8 or WAI-12 scores, suggesting that diagnostic categories did not substantially shape clients' perceptions of satisfaction or alliance. Similarly, therapy preference showed no significant differences in either outcome. However, the regression models that included multiple predictors were significant for both CSQ-8 (F = 5.985, *P* < .001) and WAI-12 (F = 2.425, *P* = .008), indicating that the combination of factors collectively accounted for meaningful variance in satisfaction and alliance. These findings underscore the complex interplay of variables that influence therapeutic outcomes and highlight the importance of therapy type in determining client satisfaction.

**Table 9:** ANOVA and regression results – influence of diagnosis, therapy type, preference, and combined predictors on CSQ-8 and WAI-12 scores, *n* = 377

Comparison	The sum of squares (between groups)	df	Mean square	F value	*P* value (Sig.)
**CSQ-8 × General Diagnosis**	9.576	1	9.576	0.401	0.527
**WAI-12 × General Diagnosis**	0.053	1	0.053	0.000	0.983
**CSQ-8 × Specific Diagnosis**	168.606	5	33.721	1.424	0.215
**WAI-12 × Specific Diagnosis**	655.680	5	131.136	1.186	0.316
**CSQ-8 × Therapy Type**	420.659	2	210.330	9.218	0.000
**WAI-12 × Therapy Type**	615.877	2	307.939	2.804	0.062
**CSQ-8 × Therapy Preference**	109.572	2	54.786	2.317	0.100
**WAI-12 × Therapy Preference**	373.976	2	186.988	1.693	0.185
**CSQ-8 Regression Model**	1258.420	10	125.842	5.985	0.000
**WAI-12 Regression Model**	2590.550	10	259.055	2.425	0.008

Table 10 displays the regression analysis for CSQ-8 scores, showing that gender, marital status, specific diagnosis, and therapy type were significant predictors, with therapy type being the strongest contributor (*P* < .001). In contrast, WAI-12 scores were significantly influenced by employment status and therapy type, with therapy type again emerging as a meaningful predictor. Other variables, such as age group, education level, and therapy preference, did not show significant predictive value in either model. These findings suggest that specific demographic and clinical variables selectively influence client satisfaction and the therapeutic alliance, underscoring the importance of tailoring interventions to these predictors.

**Table 10:** Regression coefficients for predictors of CSQ-8 and WAI-12 scores among study participants, *n* = 377

Predictor	CSQ-8 B	SE	Beta	*t*	*P*	WAI-12 B	SE	Beta	*t*	*P*
**Constant**	14.941	3.547	—	4.213	0.000	34.004	7.994	—	4.253	0.000
**Gender**	1.593	0.546	0.150	2.919	0.004	2.215	1.230	0.097	1.801	0.073
**Marital status**	1.331	0.640	0.160	2.078	0.038	1.722	1.444	0.096	1.193	0.234
**Age group**	0.231	0.319	0.062	0.722	0.471	-0.460	0.720	-0.057	-0.639	0.523
**Education level**	0.078	0.390	0.010	0.199	0.842	0.231	0.879	0.014	0.263	0.793
**Employment status**	-0.229	0.223	-0.064	-1.028	0.305	-1.076	0.503	-0.140	-2.138	0.033
**Residency area**	0.128	0.135	0.047	0.944	0.346	0.256	0.305	0.044	0.840	0.401
**General diagnosis**	1.583	1.067	0.159	1.484	0.139	2.593	2.404	0.121	1.078	0.282
**Specific diagnosis**	0.654	0.314	0.222	2.080	0.038	0.972	0.708	0.153	1.371	0.171
**Therapy type**	1.161	0.300	0.200	3.869	0.000	1.704	0.676	0.136	2.520	0.012
**Preference**	0.002	0.307	0.000	0.008	0.994	-0.605	0.692	-0.047	-0.874	0.383
Constant (WAI)	34.004	7.994	—	4.253	0.000	Significant				
Therapy Type (WAI)	1.704	0.676	0.136	2.520	0.012	Significant				

**Figure 3:**
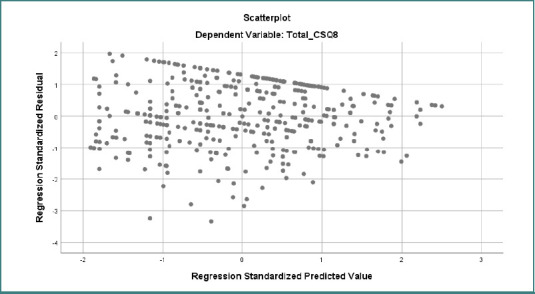
Standardized residual scatterplot for the dependent variable Total_CSQ8. The pattern suggests a slight funnel shape, suggesting potential heteroscedasticity that may affect the assumption of equal variance in regression analysis.

The scatterplot of standardized residuals versus standardized predicted values for the dependent variable Total_CSQ8 demonstrates that the assumptions of linear regression were largely satisfied (Figure 3). The residuals appeared to be randomly distributed and symmetrically around the zero line, with no apparent patterns or curves, suggesting that the linearity assumption was met. The spread of residuals remained relatively constant across predicted values, indicating homoscedasticity (equal variance). While a few data points fell beyond the ±3 range, potentially indicating outliers, they did not appear to form a systematic deviation. Overall, the scatterplot supports the adequacy of the regression model, and no significant violations of assumptions were observed.

**Figure 4:**
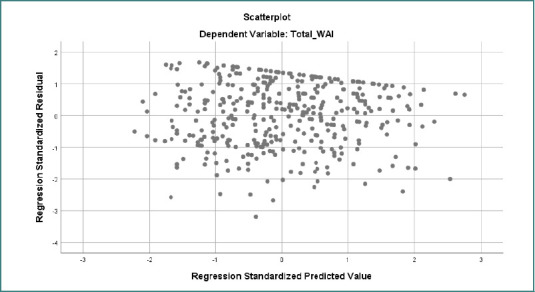
Scatterplot of standardized residuals against standardized predicted values for the dependent variable (Total_WAI). This plot assesses the assumption of homoscedasticity in the regression analysis. The residuals appear randomly dispersed around zero, indicating that the assumption of equal variance (homoscedasticity) is reasonably met.

The scatterplot of standardized residuals versus standardized predicted values for the dependent variable Total _WAI shows a generally random distribution of residuals around zero, suggesting that the assumption of linearity was reasonably satisfied (Figure 4). The variance of the residuals appeared consistent across the range of predicted values, supporting the assumption of homoscedasticity. However, a slight funnel-shaped pattern with marginally reduced spread at higher predicted values may indicate minor heteroscedasticity. There was no clear curvilinear pattern, suggesting that nonlinearity was not a significant concern. A few outliers were visible beyond ±3, but they did not seem to affect the overall distribution. In conclusion, the scatterplot provides adequate evidence for the validity of the linear regression model for Total _WAI, with only minimal concerns.

## DISCUSSION

The present study examined the therapeutic alliance and client satisfaction across teletherapy, in-person, and hybrid therapy formats among individuals with depression and anxiety disorders. Findings indicated that while in-person therapy fostered the strongest therapeutic alliances, both teletherapy and hybrid formats achieved similar levels of client satisfaction. This suggests a growing acceptance of remote care and highlights the adaptability of digital formats in contemporary psychotherapy practice [31,32].

These results align with recent studies indicating that teletherapy can yield outcomes comparable to face-to-face therapy when therapists maintain empathetic engagement and structured communication. Linardon *et al*. [32] found in a meta-analysis that therapeutic alliance scores did not significantly differ between telehealth and traditional therapy for clients with mood and anxiety disorders [31]. Similarly, Norwood *et al*. reported that videoconferencing psychotherapy maintains comparable alliance quality and treatment outcomes when therapists employ deliberate rapport-building strategies [33].

In Saudi Arabia, studies have shown growing public acceptance of teletherapy. Alghamdi *et al*. reported that clients receiving online psychotherapy for anxiety and depression expressed high satisfaction levels, citing convenience, privacy, and reduced stigma [34]. However, some participants in the current study reported challenges in establishing emotional closeness during virtual sessions, consistent with previous research emphasizing the importance of nonverbal cues and physical presence in developing a therapeutic alliance [34,35].

The therapeutic alliance remains a crucial factor in adherence and effectiveness in managing depression and anxiety, with strong alliances predicting better emotional regulation, insight, and long-term symptom improvement [36]. Nevertheless, the hybrid model—combining in-person and virtual sessions—appears to balance accessibility and relational depth, offering a flexible solution for clients facing logistical or psychological barriers to consistent therapy attendance.

Depression and anxiety continue to be significant mental health issues in Saudi Arabia, with prevalence rates ranging from 20–25% of adults [37]. According to the Saudi National Mental Health Survey (2023), anxiety disorders are the most prevalent, closely followed by depressive disorders [37]. The COVID-19 pandemic accelerated the adoption of teletherapy, with approximately 40% of mental health consultations now conducted online [38]. This expansion has been supported by governmental initiatives, such as the Saudi Vision 2030 Health Transformation Program, which emphasizes digital health access and the destigmatization of mental illness [39].

The inclusion of η^2^ indicates that therapy type had a moderate effect on client satisfaction (η^2^ ≈ 0.08–0.09), accounting for approximately 8–9% of the variance in satisfaction scores. Participants reported significantly higher satisfaction with hybrid models than with either remote or face-to-face therapy alone [31,32].

Overall, these results suggest that while satisfaction may vary modestly by demographic characteristics, the core therapeutic alliance appears resilient across diverse groups, emphasizing the universal importance of empathy, trust, and communication in achieving successful therapy outcomes [36]. Therapy modality influences client satisfaction, while the quality of the therapeutic alliance remains relatively stable regardless of diagnosis or preference.

Barriers to therapy still exist, including cultural stigma, privacy concerns, and varying levels of digital literacy, which affect engagement, particularly among older adults and those living in rural areas [40,41]. Studies in Saudi Arabia have highlighted that social and cultural perceptions can prevent individuals from fully embracing teletherapy, even when accessibility barriers are reduced, and online therapy may be perceived as less credible than in-person services [40,41]. Cultural adaptation in therapy, such as incorporating local idioms and culturally validated tools, such as the Mental Illness Associated Stigma (MIAS) scale, is critical for enhancing acceptance and effectiveness [42,43].

The findings have practical implications for mental health professionals and service designers. Clinicians should focus on enhancing the delivery of teletherapy, ensuring it is both technically smooth and relationally engaging. Following APA telepsychology guidelines—securing client privacy, preparing clients for virtual sessions, and monitoring nonverbal cues—can significantly enhance client experience [44]. As diagnosis does not substantially impact outcomes, clinicians should emphasize common therapeutic factors such as empathy, collaboration, and shared goal setting [44]. Since therapy preference did not reliably predict outcomes, clients may benefit from guided experimentation with therapy modalities rather than rigid preference matching [32]. Additionally, demographic variables suggest the need for tailored engagement strategies, including feedback-informed therapy or flexible scheduling options, particularly for under-engaged groups [34,44].

In sum, this study underscores the importance of adopting flexible, culturally sensitive, and evidence-based approaches to delivering psychotherapy in diverse, digitally evolving healthcare settings, with hybrid therapy models offering an emerging solution [31-44].

## Conclusion

The research revealed that Saudi clients dealing with depression and anxiety expressed the highest satisfaction with hybrid therapy, indicating that a mix of in-person and online sessions strikes an ideal balance between accessibility and the depth of the therapeutic relationship. Although the therapeutic alliance was consistently strong across all therapy formats, factors such as education and employment status were significantly associated with its quality, suggesting that personal background can affect therapeutic engagement. Despite a preference for face-to-face therapy among many participants, a considerable number received teletherapy, pointing to a discrepancy between client preferences and the services provided. Notably, the treatment modality emerged as the most significant predictor of satisfaction, rather than diagnosis or preference. At the same time, the therapeutic alliance remained stable when best practices were adhered to. These results highlight the importance of culturally sensitive, adaptable, and preference-informed care models and advocate for integrating hybrid therapy formats to improve the effectiveness and accessibility of mental health services in Saudi Arabia.

### Strengths and limitations

Strengths of the study include the use of validated Arabic instruments, a nationwide sample, and the concurrent evaluation of satisfaction and alliance. However, limitations include self-selection bias towards young, urban females, limited representation of rural populations, reliance on cross-sectional data and snowball sampling, and gender imbalance, which limit generalizability and the ability to infer causality.

### Recommendations and practical implications

1. Mental health facilities should focus on integrating hybrid therapy formats, as they enhance satisfaction by merging the benefits of both face-to-face and teletherapy methods. This adaptability can better meet clients' diverse needs, preferences, and lifestyles.
2. Mental health services should use screening tools or intake protocols to determine clients' preferred therapy methods and aim to match them accordingly. Addressing the mismatch between preferred and received therapy formats can boost satisfaction and engagement.
3. Enhance therapist training for digital competence through ongoing professional development programs that should emphasize building therapists' skills in providing high-quality virtual care, including maintaining therapeutic relationships, ensuring confidentiality, and using culturally sensitive communication techniques in online environments.
4. Given the link between education, employment, and the quality of therapeutic relationships, therapists should be trained to adjust their strategies more effectively to support clients with diverse educational and employment backgrounds, thereby fostering more inclusive therapeutic relationships.
5. Addressing privacy and confidentiality in teletherapy through institutions. Establishing clear guidelines and secure platforms is crucial to ensure client privacy in teletherapy, particularly considering regional cultural sensitivities, thereby fostering trust and promoting comfort during virtual sessions.
6. Future research should include qualitative studies to investigate the underlying reasons behind client preferences, perceived barriers, and cultural influences that shape satisfaction and alliance in various therapy formats. Longitudinal studies should track alliance trajectories across modalities and explore whether teletherapy-specific competencies mitigate the modest relational gap observed in this context. Research targeting rural and older populations is also warranted to assess equity of access and outcomes.

### Practical implications

These implications emphasize the need for Saudi Arabia's mental health systems to evolve by embracing technology while maintaining sensitivity to cultural values and client preferences.

The Vision 2030 Health Transformation Program and the country's current healthcare infrastructure can be used to integrate hybrid therapy into Saudi mental health services. Clinics can use a flexible scheduling system in which follow-up or less involved sessions take place via secure telehealth platforms, while complex sessions or initial assessments are done in person. Standardized protocols for telepsychology can be implemented by hospitals and primary care facilities, guaranteeing patient privacy, data security, and care continuity. The following infrastructure investments can help healthcare policy frameworks facilitate this integration:

- The provision of high-speed internet, secure digital platforms, and electronic health record integration.
- Workforce development, including teaching medical professionals culturally sensitive techniques, hybrid care coordination, and telepsychology.
- Policies to ensure hybrid therapy sessions are reimbursed equivalently to in-person care and follow standardized clinical protocols.
- Regulations to guarantee that sessions of hybrid therapy are paid on par with in-person care and adhere to established clinical protocols.

## Data Availability

Further data is available from the corresponding author upon reasonable request.

## Personal thanks

The researchers would like to acknowledge the participants' support and cooperation in this study. The researchers also express gratitude to Dr. Neama Hantira for her guidance and support in data analysis. Special thanks to Ms. Boshra Alammari and Ms. Joud Alhussini for their help in data collection procedure, as well as to the educational institutions and Psychological centers for facilitating the data collection process and providing the necessary resources for the study.
